# Heterozygous *POLG* variant Ser1181Asn co-segregating in a family with autosomal dominant axonal neuropathy, proximal muscle fatigability, ptosis, and ragged red fibers

**DOI:** 10.1186/s42466-022-00169-w

**Published:** 2022-02-01

**Authors:** Maike F. Dohrn, Corina Heller, Diana Zengeler, Carolin D. Obermaier, Saskia Biskup, Joachim Weis, Stefan Nikolin, Kristl G. Claeys, Ulrike Schöne, Danique Beijer, Natalie Winter, Pascal Achenbach, Burkhard Gess, Jörg B. Schulz, Lejla Mulahasanovic

**Affiliations:** 1 Department of Neurology, Medical Faculty, RWTH Aachen University, Aachen, Germany; 2 Dr. John T. Macdonald Foundation, Department of Human Genetics and John P. Hussman Institute for Human Genomics, University of Miami, Miller School of Medicine, Miami, FL USA; 3 Praxis Für Humangenetik Tübingen, Tuebingen, Germany; 4 CeGaT GmbH, Tuebingen, Germany; 5 Institute of Neuropathology, Medical Faculty, RWTH Aachen University, Aachen, Germany; 6 Department of Neurology, University Hospitals Leuven, Leuven, Belgium; 7 Laboratory for Muscle Diseases and Neuropathies, KU Leuven, Leuven, Belgium; 8 Department of Neurology and Epileptology, Hertie Institute for Clinical Brain Research, University of Tuebingen, Tuebingen, Germany; 9 JARA-BRAIN Institute Molecular Neuroscience and Neuroimaging, Forschungszentrum Jülich GmbH and RWTH Aachen University, Aachen, Germany

**Keywords:** Polymerase gamma, Autosomal dominant, Axonal neuropathy, Myo-neuropathy, Mitochondrial myopathy

## Abstract

By whole-exome sequencing, we found the heterozygous *POLG* variant c.3542G>A; p.Ser1181Asn in a family of four affected individuals, presenting with a mixed neuro-myopathic phenotype. The variant is located within the active site of polymerase gamma, in a cluster region associated with an autosomal dominant inheritance. In adolescence, the index developed distal atrophies and weakness, sensory loss, afferent ataxia, double vision, and bilateral ptosis. One older sister presented with Charcot-Marie-Tooth-like symptoms, while the youngest sister and father reported exercise-induced muscle pain and proximal weakness. In none of the individuals, we observed any involvement of the central nervous system. Muscle biopsies obtained from the father and the older sister showed ragged-red fibers, and electron microscopy confirmed mitochondrial damage. We conclude that this novel *POLG* variant explains this family’s phenotype.

## Case report

In her teenage years, the currently 57-year-old female index patient noticed progressive gait instability, distal muscle weakness, and distal sensory deficits. Fine motor skills worsened, and her vision became remotely doubled with fatigability. Clinically, she showed an advanced distal tetraparesis including steppage gait, sensory deficits on lower legs, afferent ataxia, distal areflexia, and a slight bilateral non-dynamic ptosis. Nerve conduction studies (NCS) confirmed a severe chronic axonal sensorimotor polyneuropathy. A detailed patient history and repeated laboratory tests did not reveal any acquired cause of neuropathy.

**Fig. 1:**
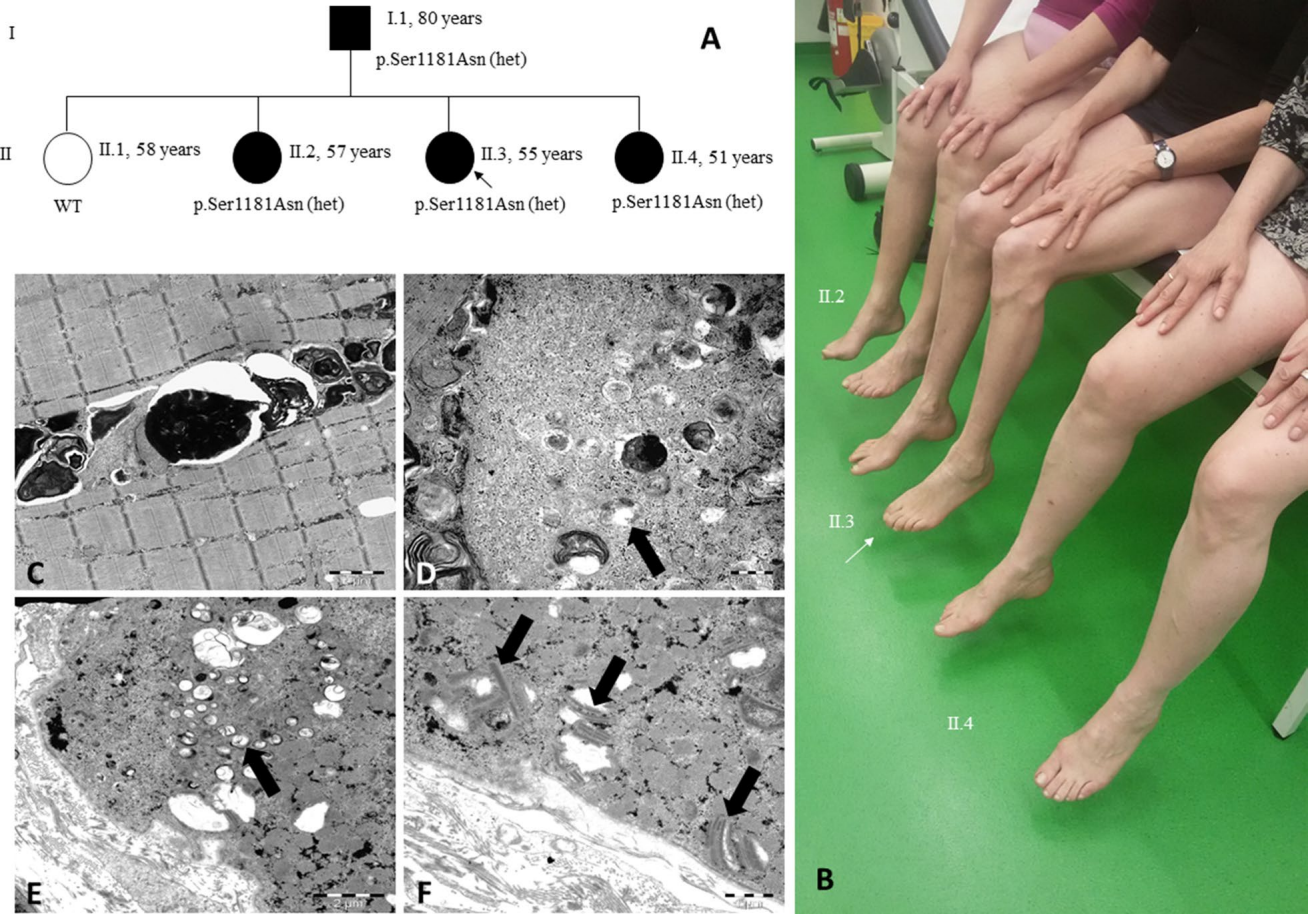
Pedigree, clinical picture, and electron microscopy. In a family with an affected father, three affected and one unaffected sisters, the novel *POLG* variant c.3542G>A; p.Ser1181Asn co-segregates with the phenotype in two generations (**a**). Clinical features are distal atrophies and high-arched feet in the first (II.2) and second (II.3) oldest affected sister sitting on the right side and in the middle of the bench (**b**). The youngest sister (II.4) has a pronounced myopathic phenotype. An electron microscopy of the lateral vastus muscle obtained from patient II.2 revealed prominent intermyofibrillar autophagic vacuoles filled with pleomorphic granular and membranous material (**c**), degenerating mitochondria undergoing (abnormal) mitophagy (arrows; **d, e**), and paracrystalline inclusions in many mitochondria (arrows, **f**), demonstrating mitochondrial damage

The index patient (II.3) had two older and one younger sister, of whom the second (II.2) and fourth (II.4) born had neuromuscular symptoms as well, while the oldest sister (II.1) was unaffected (Fig. 1a). Patient II.2 presented with moderate weakness and atrophies in lower legs, distal sensory deficits, hyporeflexia, and pedes cavi. Patient II.4 reported proximal muscle weakness, exercise-induced muscle pain, and cramping. She had previously undergone surgery for bilateral ptosis. Their father (I.1) suffered from proximal muscle weakness and atrophies. His serum creatine kinase was moderately elevated (around 600 U/l; normal < 190 U/l), which was not the case in any of his daughters. NCS revealed an axonal sensorimotor polyneuropathy in patient I.1 and II.2, but not in patient II.4. We did not observe any significant decrement in the 3 Hz repetitive muscle stimulation. An electromyogram performed in patient I.1 at the left tibial anterior muscle showed a mixed neuropathic and myopathic pattern.

Whole-exome sequencing performed in patients II.2 and II.3 revealed the heterozygous variant c.3542G>A; p.Ser1181Asn in *POLG* (NM_001126131.3). Sanger sequencing-based segregation analyses confirmed that all affected family members carried the variant, whereas the unaffected sister (II.1) did not (Fig. 1a, b). A muscle biopsy obtained from patient II.2’s right lateral vastus muscle showed signs of denervation as well as a combination of COX-negative and ragged red fibers, which had previously been reported in the father’s muscle as well (analysis performed elsewhere). Additionally, the electron microscopy revealed degenerating mitochondria with para-crystalline inclusions as well as mitochondria undergoing impaired mitophagy (Fig. 1c–f).

## Discussion

Polymerase gamma (*POLG*) is a 140 kDa enzyme responsible for mitochondrial DNA replication [3]. The spectrum of *POLG*-associated phenotypes is broad, and there is currently no disease-modifying treatment available. With an autosomal recessive mode of inheritance, mtDNA depletion syndromes such as Alpers-Huttenlocher syndrome cause severe encephalopathies with epilepsy, ataxia, parkinsonism, and mental retardation with an onset at early childhood [10]. Autosomal dominant disease forms are caused by variants clustering within the DNA-binding palm- and finger-domains [1] and typically manifest with a variable form of external ophthalmoplegia [4], frequently accompanied by generalized myopathy, tremor, or parkinsonism [5]. Axonal neuropathies are part of the known *POLG* spectrum [7, 12], typically with a sensory-ataxic phenotype [6]. It is further known that the age of onset and the severity of symptoms can vary even within carriers of dominant *POLG* mutations [2], like in the herein reported family.

Co-segregating in a pedigree of autosomal dominant inheritance, we herein report the heterozygous *POLG* variant c.3542G>A; p.Ser1181Asn, for the first time in association with any disease. The variant is located within a hot spot region encoding the polymerase active site: “Holding” the template DNA strand, the so-called palm domain together with the finger and thumb domains are crucial for mitochondrial DNA replication [3, 8, 11]. Functional studies on the variant p.Tyr955Cys, likewise associated with a dominant mode of inheritance [5], showed a significantly reduced nucleotide incorporation rate with a higher amount of replication errors [9]. *In-silico* predictions for the novel variant c.3542G>A; p.Ser1181Asn consistently support its pathogenicity. In the gnomAD population database, the overall allele frequency is 0.00003182, with a total of nine heterozygotes, which might be explained by a reduced penetrance, a phenomenon that has been previously described for other pathogenic *POLG* variants.

We conclude that the herein described heterozygous variant c.3542G>A; p.Ser1181Asn in *POLG* is likely to explain the patients’ phenotype. In families with a variable neuro-myopathic syndrome and autosomal dominant inheritance, we would consider *POLG* as a fitting molecular genetic cause.

## Data Availability

Not applicable.

## Abbreviations

CK: Creatine kinase
NCS: Nerve conduction studies
POLG: Polymerase G
